# Detailed clinical features and genotype–phenotype correlation in an *OTOF*-related hearing loss cohort in Japan

**DOI:** 10.1007/s00439-021-02351-7

**Published:** 2021-09-18

**Authors:** Yoh-ichiro Iwasa, Shin-ya Nishio, Hidekane Yoshimura, Akiko Sugaya, Yuko Kataoka, Yukihide Maeda, Yukihiko Kanda, Kyoko Nagai, Yasushi Naito, Hiroshi Yamazaki, Tetsuo Ikezono, Han Matsuda, Masako Nakai, Risa Tona, Yuika Sakurai, Remi Motegi, Hidehiko Takeda, Marina Kobayashi, Chiharu Kihara, Takashi Ishino, Shin-ya Morita, Satoshi Iwasaki, Masahiro Takahashi, Sakiko Furutate, Shin-ichiro Oka, Toshinori Kubota, Yasuhiro Arai, Yumiko Kobayashi, Daisuke Kikuchi, Tomoko Shintani, Noriko Ogasawara, Yohei Honkura, Shuji Izumi, Misako Hyogo, Yuzuru Ninoyu, Mayumi Suematsu, Jun Nakayama, Nana Tsuchihashi, Mayuri Okami, Hideaki Sakata, Hiroshi Yoshihashi, Taisuke Kobayashi, Kozo Kumakawa, Tadao Yoshida, Tomoko Esaki, Shin-ichi Usami

**Affiliations:** 1 Department of Otorhinolaryngology, Shinshu University School of Medicine, Matsumoto, Japan; 2 Department of Hearing Implant Sciences, Shinshu University School of Medicine, 3-1-1, Asahi, Matsumoto City, 390-8621 Japan; 3 Department of Otolaryngology-Head and Neck Surgery, Okayama University Graduate School of Medicine, Dentistry and Pharmaceutical Sciences, Okayama, Japan; 4 Kanda ENT Clinic, Nagasaki Bell Hearing Center, Nagasaki, Japan; 5 TAKASAKI Ear Nose and Throat Clinic, Takasaki, Japan; 6 Department of Otolaryngology, Kobe City Medical Center General Hospital, Kobe, Japan; 7 Department of Otorhinolaryngology, Saitama School of Medicine, Moroyama, Japan; 8 Shiga Medical Center for Children, Shiga, Japan; 9 Department of Otorhinolaryngology, Jikei University School of Medicine, Tokyo, Japan; 10 Department of Otorhinolaryngology, Juntendo University Faculty of Medicine, Tokyo, Japan; 11 Department of Otorhinolaryngology, Toranomon Hospital, Tokyo, Japan; 12 Department of Otolaryngology-Head and Neck Surgery, Nagasaki University Graduate School of Biomedical Sciences, Nagasaki, Japan; 13 Department of Otorhinolaryngology, Head and Neck Surgery, Hiroshima University Hospital, Hiroshima, Japan; 14 Department of Otolaryngology-Head and Neck Surgery, Faculty of Medicine and Graduate School of Medicine, Hokkaido University, Sapporo, Japan; 15 Department of Otorhinolaryngology, International University of Health and Welfare, Mita Hospital, Tokyo, Japan; 16 Department of Otolaryngology, Head and Neck Surgery, Yamagata University Faculty of Medicine, Yamagata, Japan; 17 Department of Otorhinolaryngology-Head and Neck Surgery, Yokohama City University School of Medicine, Yokohama, Japan; 18 Department of Otolaryngology-Head and Neck Surgery, Iwate Medical University, Morioka, Japan; 19 Department of Otolaryngology, Fukushima Medical University, Fukushima, Japan; 20 Department of Microbiology, Sapporo Medical University School of Medicine, Sapporo, Japan; 21 Department of Otolaryngology-Head and Neck Surgery, Tohoku University School of Medicine, Sendai, Japan; 22 Department of Otolaryngology Head and Neck Surgery, Niigata University Graduate School of Medical and Dental Sciences, Niigata, Japan; 23 Department of Otolaryngology-Head and Neck Surgery, Kyoto Prefectural University of Medicine, Kyoto, Japan; 24 Department of Otorhinolaryngology, Shiga University School of Medical Science, Otsu, Japan; 25 Department of Otorhinolaryngology, Graduate School of Medical Sciences, Kyushu University, Fukuoka, Japan; 26 Department of Otorhinolaryngology, Tokai University School of Medicine, Isehara, Japan; 27 Kawagoe Otology Institute, Kawagoe, Japan; 28 Department of Medical Genetics, Tokyo Metropolitan Children’s Medical Center, Tokyo, Japan; 29 Department of Otolaryngology, Kochi University Medical School, Kochi, Japan; 30 Department of Otolaryngology, Kamio Memorial Hospital, Tokyo, Japan; 31 Department of Otorhinolaryngology, Nagoya University Graduate School of Medicine, Nagoya, Japan; 32 Department of Otolaryngology, Aichi Children’s Health and Medical Center, Obu, Japan

## Abstract

Mutations in the *OTOF* gene are a common cause of hereditary hearing loss and the main cause of auditory neuropathy spectrum disorder (ANSD). Although it is reported that most of the patients with *OTOF* mutations have stable, congenital or prelingual onset severe-to-profound hearing loss, some patients show atypical clinical phenotypes, and the genotype–phenotype correlation in patients with *OTOF* mutations is not yet fully understood. In this study, we aimed to reveal detailed clinical characteristics of *OTOF*-related hearing loss patients and the genotype–phenotype correlation. Detailed clinical information was available for 64 patients in our database who were diagnosed with *OTOF*-related hearing loss. As reported previously, most of the patients (90.6%) showed a “typical” phenotype; prelingual and severe-to-profound hearing loss. Forty-seven patients (73.4%) underwent cochlear implantation surgery and showed successful outcomes; approximately 85–90% of the patients showed a hearing level of 20–39 dB with cochlear implant and a Categories of Auditory Performance (CAP) scale level 6 or better. Although truncating mutations and p.Arg1939Gln were clearly related to severe phenotype, almost half of the patients with one or more non-truncating mutations showed mild-to-moderate hearing loss. Notably, patients with p.His513Arg, p.Ile1573Thr and p.Glu1910Lys showed “true” auditory neuropathy-like clinical characteristics. In this study, we have clarified genotype–phenotype correlation and efficacy of cochlear implantation for *OTOF*-related hearing loss patients in the biggest cohort studied to date. We believe that the clinical characteristics and genotype–phenotype correlation found in this study will support preoperative counseling and appropriate intervention for *OTOF*-related hearing loss patients.

**Supplementary Information:**

The online version contains supplementary material available at 10.1007/s00439-021-02351-7.

## Introduction

Hearing loss is one of the most common sensory disorders, with around 466 million people suffering from hearing loss (World Health Organization 2021). In developed countries, it is reported that 1 out of 500 newborns has bilateral hearing loss and approximately 80% of cases are due to genetic etiologies (Shearer et al. 2017). *OTOF* is reported to be the causative gene of DFNB9 and one of the common causes of non-syndromic recessive sensorineural hearing loss. The prevalence of *OTOF* mutations has been reported to be 1.4–3.2% of non-syndromic hearing loss cases (Rodríguez-Ballesteros et al. 2008; Choi et al. 2009; Wang et al. 2010a; Mahdieh et al. 2012) and 1.72% in Japan (Iwasa et al. 2019). To date, more than 220 mutations in *OTOF* have been reported (Vona et al. 2020). Although it is reported that most of the patients with *OTOF* mutations have stable, congenital or prelingual onset severe-to-profound hearing loss, some patients show atypical clinical phenotypes, such as mild-to-moderate progressive cases (Chiu et al. 2010). The genotype–phenotype correlation in the patients with *OTOF* mutations is, subsequently, not yet fully understood.

*OTOF* is also reported to be a main cause of auditory neuropathy spectrum disorder (ANSD), which is a specific form of hearing loss with an abnormal auditory brainstem response (ABR) and the presence of otoacoustic emissions (OAEs) (Starr et al. 1996). Both genetic and environmental factors cause ANSD, such as hyperbilirubinemia, thiamine deficiency, hypoxia, and noise-induced and age-related hearing loss (Shearer 2019). *OTOF*-related ANSD is the most prevalent form of ANSD, and it is reported that 23–90.9% of pediatric cases of ANSD are caused by *OTOF* mutations (Rodríguez-Ballesteros et al. 2008; Matsunaga et al. 2012; Zhang et al. 2016; Kim et al. 2018). *OTOF* is mainly expressed in the inner hair cells (IHCs), and mutations in the *OTOF* gene cause dysfunction of synaptic exocytosis at the ribbon synapse (Roux et al. 2006). *OTOF*-related ANSD is also known as auditory synaptopathy, presenting with impaired synaptic function between inner hair cells and spiral ganglion neurons, to differentiate it from auditory neuropathy (i.e., “true” auditory neuropathy) (Moser and Starr 2016). Due to the presence of an OAE response, there are some clinical problems with ANSD patients; they inappropriately pass the newborn hearing screening (NBHS) in countries where OAE is used as a screening tool, resulting in the late diagnosis of hearing loss. As early intervention by hearing aid (HA), cochlear implant (CI) and language rehabilitation are necessary for patients with hearing impairment to develop their speech skills, late diagnosis is a disadvantage for such hearing loss patients. Moreover, the presence of an OAE response makes it difficult for clinicians to decide the appropriate timing of cochlear implantation, as OAE positivity means the outer hair cells (OHCs) are functional and the efficacy of CI could be limited in cases in which the patient has “true” auditory neuropathy. Therefore, the genetic diagnosis of *OTOF*-related hearing loss in ANSD patients can encourage clinicians to recommend cochlear implantation for the patients, as the performance of CI for *OTOF*-related ANSD is reported to be excellent (Zheng and Liu 2020). However, each report was based on only a limited number of patients, and different evaluation tools were used for assessing the performance of CIs in each report.

In Japan, social health insurance-based genetic testing for the patients with hereditary hearing loss was approved in 2012 and, from 2015, massively parallel DNA sequencing technology was combined with genetic testing, so that excellent mutation data from Japanese hearing loss patients has been accumulated over the years in the whole-Japanese database. In this study, we investigated the clinical data of the patients diagnosed with *OTOF*-related hearing loss registered in our database, and aimed to reveal detailed clinical characteristics of *OTOF*-related hearing loss patients and the genotype–phenotype correlation in the patients with *OTOF* mutations.

## Subjects and methods

### Subjects

A total of 12,137 patients were registered in our database between February 2012 and December 2020 from 96 otolaryngology departments from across Japan. Seventy-four patients had two or more mutations in the *OTOF* gene. Detailed clinical information was available for 66 of 74 patients, and their clinical characteristics were analyzed retrospectively through the review of their medical records, and 2 of them were excluded because the clinical phenotype was completely incompatible with *OTOF*-related hearing loss: One of them had two mutations of uncertain significance and this case had late-onset ski-slope hearing loss, with the other having three mutations of uncertain significance and acute-onset unilateral mild hearing loss. Hearing level was evaluated using pure-tone audiometry (PTA) classified by a pure-tone average over 500, 1000, 2000 and 4000 Hz in the better hearing ears. For children who could not undergo PTA, we used an average over 500, 1000, 2000 and 4000 Hz in conditioned oriented reflex audiometry (COR). Severity of hearing loss was classified as follows; normal hearing, < 25 dB; mild hearing loss, 25–39 dB; moderate hearing loss, 40–69 dB; severe hearing loss, 70–89 dB; and profound hearing loss, greater than 90 dB. Written informed consent was obtained from all subjects (or from their next of kin, caretaker, or guardian on the behalf of minors/children) prior to enrollment in the project. This study was approved by the ethical committees of Shinshu University and each of the other participating institutions.

## MPS sequencing

### Amplicon resequencing with MPS

Amplicon libraries were prepared using an Ion AmpliSeq Custom Panel (Applied Biosystems, Life Technologies), in accordance with the manufacturer’s instructions, for 68 genes reported to cause non-syndromic hereditary HL. The detailed sample preparation protocol has been described elsewhere (Iwasa et al. 2019). Sequencing was performed in accordance with the manufacturer’s instructions. Massively Parallel Sequencing (MPS) was performed with an Ion Torrent Personal Genome Machine (PGM) system, Ion Proton System or Ion S5 system using an Ion PGM 200 Sequencing Kit with an Ion 318 Chip (Life Technologies) or Ion HiQ Chef Kit with an Ion P1 chip or Ion 540 Chip kit-Chef. The sequence data were mapped against the human genome sequence (build GRCh37/hg19) with a Torrent Mapping Alignment Program. After sequence mapping, the DNA variants were detected with Torrent Variant Caller plug-in software. After variant detection, their effects were analyzed using ANNOVAR software (Wang et al. 2010b). The missense, nonsense, insertion/deletion, and splicing variants were selected from among the identified variants. Variants were further selected as less than 1% of (1) the 1000 genome database, (2) the 6500 exome variants, (3) the Human Genetic Variation Database (dataset for 1208 Japanese exome variants), and (4) the 333 in-house Japanese normal hearing controls by using our database software. All the mutations found in this study were confirmed by Sanger sequencing using exon-specific custom primers. The pathogenicity of the candidate variants was interpreted based on the standards and guidelines of the American College of Medical Genetics (ACMG) and ClinGen HL-CDWG expert specification (Oza et al. 2018; Brandt et al. 2019).

## Results

### Mutation analysis

Detailed mutational information from 64 patients whose clinical information was available is shown in Table 1. Twenty-seven (42.2%) of the 64 patients with biallelic *OTOF* mutations had homozygous *OTOF*: NM_001287489:c.5816G > A: p.Arg1939Gln mutations. Twenty-nine (45.3%) were compound heterozygote with p.Arg1939Gln and another mutation. Eight patients (12.5%) had other non- p.Arg1939Gln mutations. Two patients excluded from the study were found to possess compound heterozygotes with p.[Pro309Leu];[Arg813Trp] and p.[Asp217Gly];[Ala1802Val];c.[3127-5G > A], respectively. The pathogenicity of the mutations found in the study were categorized in accordance with the ACMG criteria (Suppl. Table 1). Six mutations (c.2437C > T (p.Arg813Trp), c.3126 + 5G > A, c.3570 + 5G > A, c.5838G > A (p.Trp1946Ter), c.5728G > A (p.Glu1910Lys) and c.5500delG (p.Asp1834fs)) were novel.

**Table 1:** Detailed mutational and clinical information for the cases with biallelic OTOF mutations in this study

Patient ID	Age	Mutation 1	Mutation 2	OAE*	ABR	Severity†	Intervention	CAP	Age at first CI
SNS2255	34y	p.Arg1939Gln	p.Arg1939Gln	Absent	Untested	Profound	Hearing aid	6	–
YMG2003	23y	p.Arg1939Gln	p.Arg1939Gln	NA	No response	Profound	Unilateral CI	6	5y–5y6m
2703	22y	p.Arg1939Gln	p.Arg1939Gln	NA	No response	Severe	Hearing aid	NA	–
AH6163	16y	p.Arg1939Gln	p.Arg1939Gln	NA	100–105 dB	Profound	Unilateral CI	6	6y <
SNS1171	13y	p.Arg1939Gln	p.Arg1939Gln	Present	NA	Profound	Bilateral CI (sequential)	6	1y6m–2y
AG7860	12y	p.Arg1939Gln	p.Arg1939Gln	NA	No response	Profound	Unilateral CI	6	NA
KBS5019	11y	p.Arg1939Gln	p.Arg1939Gln	Present	No response	Profound	Bilateral CI (sequential)	6	3y–3y6m
AG4113	9y	p.Arg1939Gln	p.Arg1939Gln	Present	No response	Profound	Hearing aid	3	–
OKY3003	9y	p.Arg1939Gln	p.Arg1939Gln	Present	100–105 dB	Profound	Bilateral CI (sequential)	6	1y–1y6m
SNS2292	9y	p.Arg1939Gln	p.Arg1939Gln	Present	No response	Profound	Bilateral CI (sequential)	6	1y–1y6m
AL8879	7y	p.Arg1939Gln	p.Arg1939Gln	Present	90–99 dB	Profound	Unilateral CI	5	3y6m–4y
AH1024	7y	p.Arg1939Gln	p.Arg1939Gln	NA	Untested	Profound	Bilateral CI (sequential)	6	1y6m–2y
AL8222	6y	p.Arg1939Gln	p.Arg1939Gln	Present	100–105 dB	Profound	Bilateral CI (sequential)	6	2y–2y6m
AK2367	5y	p.Arg1939Gln	p.Arg1939Gln	Present	90–99 dB	Profound	Bilateral CI (sequential)	6	1y–1y6m
AK2846	5y	p.Arg1939Gln	p.Arg1939Gln	Present	Untested	Profound	Bilateral CI (simultaneous)	7	1y–1y6m
AL8158	4y	p.Arg1939Gln	p.Arg1939Gln	Present	90–99 dB	Profound	Bilateral CI (sequential)	6	1y–1y6m
AK6678	4y	p.Arg1939Gln	p.Arg1939Gln	Present	No response	Profound	Bilateral CI (sequential)	6	1y6m–2y
AL5519	4y	p.Arg1939Gln	p.Arg1939Gln	Absent	No response	Profound	Bilateral CI (sequential)	5	1y6m–2y
AL7410	3y	p.Arg1939Gln	p.Arg1939Gln	Present	40–49 dB	Profound	Bilateral CI (simultaneous)	6	1y–1y6m
AM9889	3y	p.Arg1939Gln	p.Arg1939Gln	Present	100–105 dB	Profound	Bilateral CI (simultaneous)	6	1y6m–2y
AH0075	3y	p.Arg1939Gln	p.Arg1939Gln	Present	100–105 dB	Severe	Bilateral CI (simultaneous)	3	1y–1y6m
AM7901	2y	p.Arg1939Gln	p.Arg1939Gln	Present	No response	Profound	Unilateral CI	5	2y6m–3y
AM8170	2y	p.Arg1939Gln	p.Arg1939Gln	Present	No response	Profound	Unilateral CI	1	2y–2y6m
AK2888	1y	p.Arg1939Gln	p.Arg1939Gln	NA	No response	Profound‡	Bilateral CI (simultaneous)	NA	NA
HL9490	1y	p.Arg1939Gln	p.Arg1939Gln	Present	No response	Profound‡	Hearing aid	3	–
AK6261	1y	p.Arg1939Gln	p.Arg1939Gln	NA	Untested	Profound‡	Hearing aid	3	–
AL7917	1y	p.Arg1939Gln	p.Arg1939Gln	Present	100–105 dB	Severe‡	Hearing aid	3	–
AH1616	70y	p.Arg1939Gln	p.Tyr474Ter	NA	Untested	Severe	NA	4	–
AK9968	41y	p.Arg1939Gln	c.5533 + 1G > A	Absent	NA	Profound	Hearing aid	4	–
AH2665	32y	p.Arg1939Gln	p.Tyr474Ter	NA	Untested	Profound	Hearing aid	4	–
2958	20y	p.Arg1939Gln	p.Trp717Ter	Present	NA	Severe	Unilateral CI	6	3y6m–4y
AH6310	11y	p.Arg1939Gln	p.Gln1072Ter	NA	Untested	Profound	Bilateral CI (sequential)	7	1y6m–2y
AH1786	10y	p.Arg1939Gln	p.Tyr474Ter	Present	Untested	Profound	Bilateral CI (sequential)	6	1y6m–2y
AG6001	9y	p.Arg1939Gln	p.Tyr1064Ter	Present	No response	Profound	Bilateral CI (sequential)	6	1y–1y6m
AH8883	8y	p.Arg1939Gln	p.Pro489Ser	Present	No response	Profound	Bilateral CI (sequential)	6	2y6m–3y
AG9191	8y	p.Arg1939Gln	p.Arg1792Cys	Present	No response	NA	NA	NA	NA
AH9534	7y	p.Arg1939Gln	c.4960 + 2T > C	Absent	No response	Profound	Bilateral CI (sequential)	6	1y6m–2y
AH0904	7y	p.Arg1939Gln	p.Ile1449fs	Present	No response	Severe	Unilateral CI	6	1y–1y6m
AH6306	6y	p.Arg1939Gln	p.Gln1072Ter	Present	No response	Profound	Bilateral CI (sequential)	6	1y6m–2y
D72	6y	p.Arg1939Gln	p.Tyr1064Ter	Present	No response	Profound	Bilateral CI (sequential)	6	< 1y
AG6481	6y	p.Arg1939Gln	p.Arg1856Gln	Absent	No response	Profound	Bilateral CI (sequential)	4	1y–1y6m
AL8880	6y	p.Arg1939Gln	p.Arg1939Trp	Present	No response	Profound	Unilateral CI	6	2y–2y6m
AH0951	5y	p.Arg1939Gln	p.Tyr474Ter	Present	No response	Profound	Unilateral CI	3	1y–1y6m
AK6640	5y	p.Arg1939Gln	p.His513Arg	Untested	No response	Moderate	not-aided	6	–
AG6165	5y	p.Arg1939Gln	p.Leu1003fs	Present	NA	Profound	Bilateral CI (simultaneous)	6	1y–1y6m
AL7878	5y	p.Arg1939Gln	p.Arg897fs	Present	No response	Profound	Hearing aid	NA	–
AK2019	5y	p.Arg1939Gln	p.Arg1856Trp	Present	No response	Profound	Unilateral CI	6	NA
AL7890	4y	p.Arg1939Gln	p.Arg1939Trp	Present	90–99 dB	Profound	Bilateral CI (sequential)	6	2y6m–3y
AP8312	4y	p.Arg1939Gln	p.Glu757fs	Present	No response	Profound	Bilateral CI (sequential)	5	2y–2y6m
AM8924	3y	p.Arg1939Gln	p.Tyr474Ter	Present	No response	Profound	Bilateral CI (simultaneous)	6	1y–1y6m
AL7003	3y	p.Arg1939Gln	c.897 + 5G > A	Absent	No response	Profound	Bilateral CI (sequential)	4	1y–1y6m
AL6924	2y	p.Arg1939Gln	p.Tyr474Ter	Present	NA	Profound	Bilateral CI (simultaneous)	NA	NA
AM9635	2y	p.Arg1939Gln	p.Gln1072Ter	Present	No response	Profound	Bilateral CI (simultaneous)	0	2y–2y6m
AL7907	2y	p.Arg1939Gln	p.Trp1946Ter	Present	No response	Profound	Bilateral CI (sequential)	5	1y–1y6m
AL6094	2y	p.Arg1939Gln	p.Asp1834fs	Present	100–105 dB	Profound	Bilateral CI (sequential)	4	2y–2y6m
AL9858	1y	p.Arg1939Gln	c.3570 + 5G > A	Present	Untested	Severe‡	Hearing aid	NA	–
AL5758	18y	p.Arg1727Gln	p.1123_1129del	Absent	60–69 dB	Moderate	not-aided	NA	–
KND0018	17y	p.Arg1856Gln	p.Tyr474Ter	Present	No response	Profound	Unilateral CI	6	NA
AK3328	16y	p.Tyr474Ter	c.3126 + 5G > A	Absent	Untested	Profound	Hearing aid	3	–
AR7215	12y	p.Arg1939Trp	p.Glu1910Lys	Present	No response	Profound	Unilateral CI	NA	6y <
AK2839	10y	p.Ser247Asn	p.Glu594Lys	Untested	No response	Profound	Bilateral CI (sequential)	NA	2y6m–3y
KBS5114	9y	p.Phe1069Val	p.Ala1802Val	Untested	Untested	Moderate	Hearing aid	6	–
AL8610	9y	p.Arg1939Trp	p.Glu1910Lys	Present	No response	Moderate	Hearing aid	NA	–
AH0083	7y	p.Ile1573Thr	p.Ala1377fs	Present	No response	Moderate	Unilateral CI	6	6y <

*NA* not available, *m* month(s), *y* year(s)

^*^OAE responses are based on the results at NBHS or first testing at each institution

^†^Theresholds were determined as the average at 500, 1000, 2000 and 4000 Hz in the better hearing ear tested by PTA or COR

^‡^Hearing level tested by COR

### Clinical characteristics

**Fig. 1:**
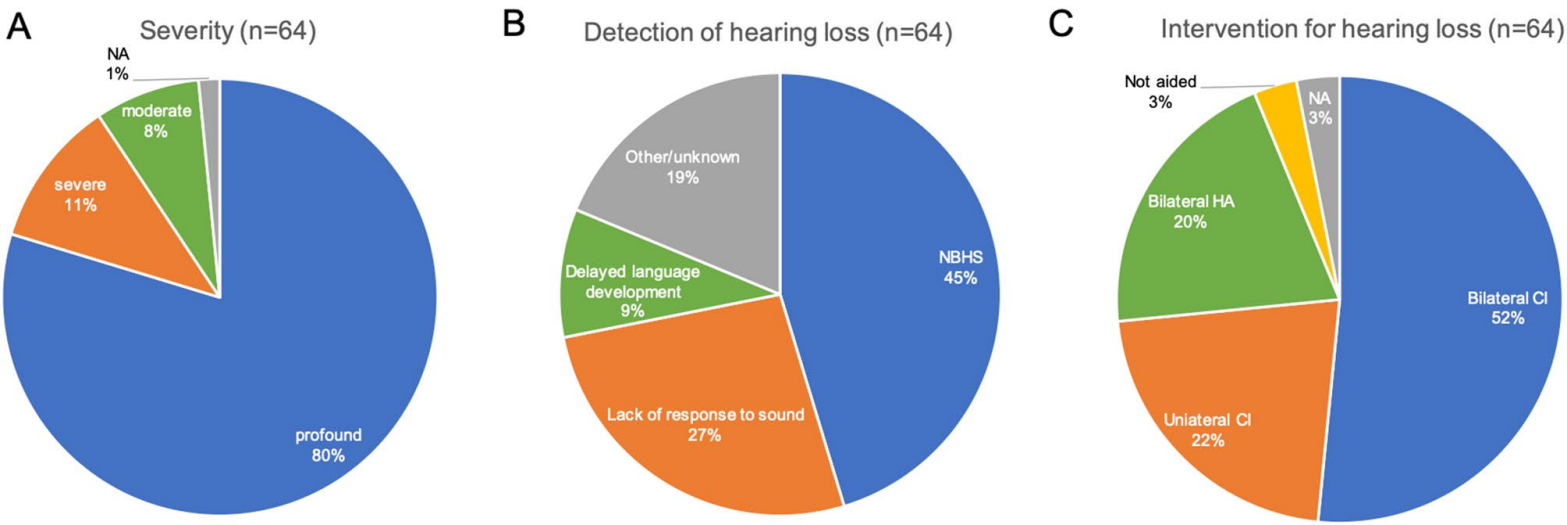
Clinical characteristics of the *OTOF*-related hearing loss patients in this study. **a** Hearing level in PTA or COR in very young children. **b** Detection of hearing loss. **c** Intervention for hearing loss. *NA* not applicable, *n* number of patients

**Fig. 2:**
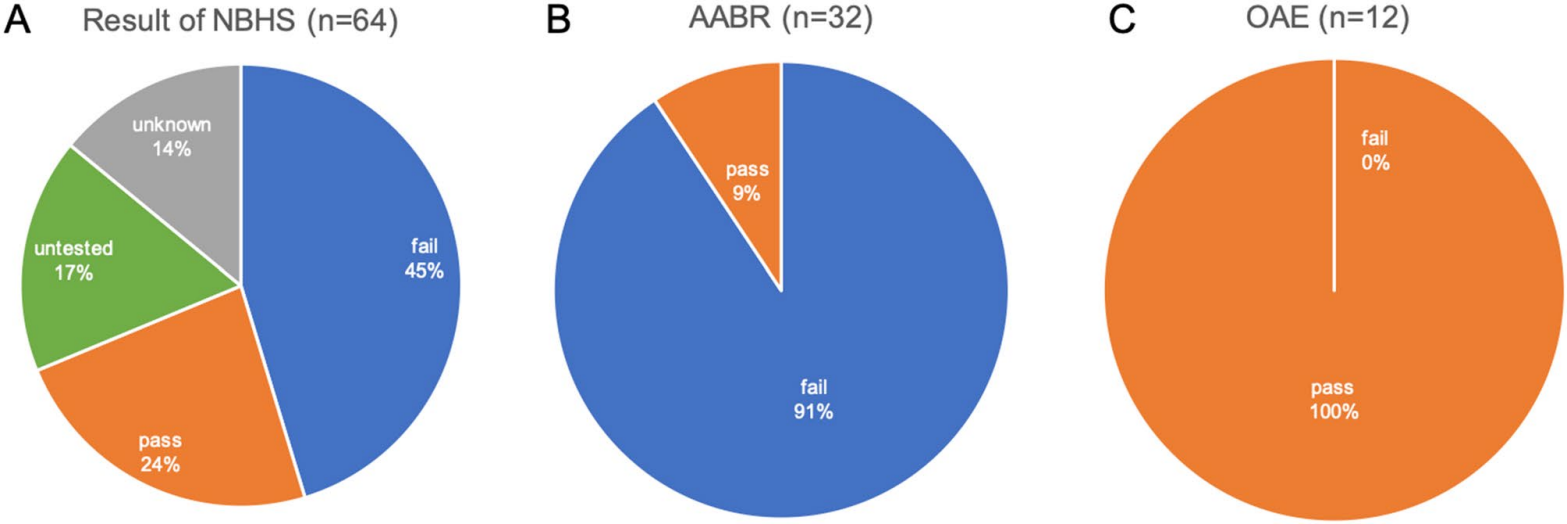
Detailed information of newborn hearing screening (NBHS). **a** Results of NBHS for all patients in this study. **b**, **c** Result of NBHS by each screening method (AABR and OAE)

A summary of clinical information is shown in Fig. 1. Although most of the patients with *OTOF*-related hearing loss show congenital severe-to-profound hearing loss (90.6%) (Fig. 1a), NBHS only detects 45.3% of these cases of hearing loss (Fig. 1b). The rest of them were found to have hearing loss due to a lack of response to sound (26.6%), delayed language development (9.4%) or other reasons. Forty-seven patients (73.4%) underwent CI surgery; 9 patients (14.1%) were implanted simultaneously in both ears, 24 of them (37.5%) were implanted sequentially and 14 of them (21.9%) were implanted unilaterally (Fig. 1c). Figure 2 shows the detailed information for NBHS; 29 patients (45.3%) failed NBHS, 14 (21.5%) passed and 12 (18.5%) did not undergo NBHS (Fig. 2a). Among the 44 patients for whom information regarding NBHS was available, the detection rate of *OTOF*-related hearing loss was 65.9% (29 out of 44 patients). AABR and OAE was performed in 32 and 12 patients, respectively. All of the 12 patients examined by OAE improperly passed NBHS, while 3 out of 32 patients (9.4%) passed AABR screening (Fig. 2b, c). All 3 cases who passed AABR screening had homozygous p.Arg1939Gln mutations.

### Performance of cochlear implantation

**Fig. 3:**
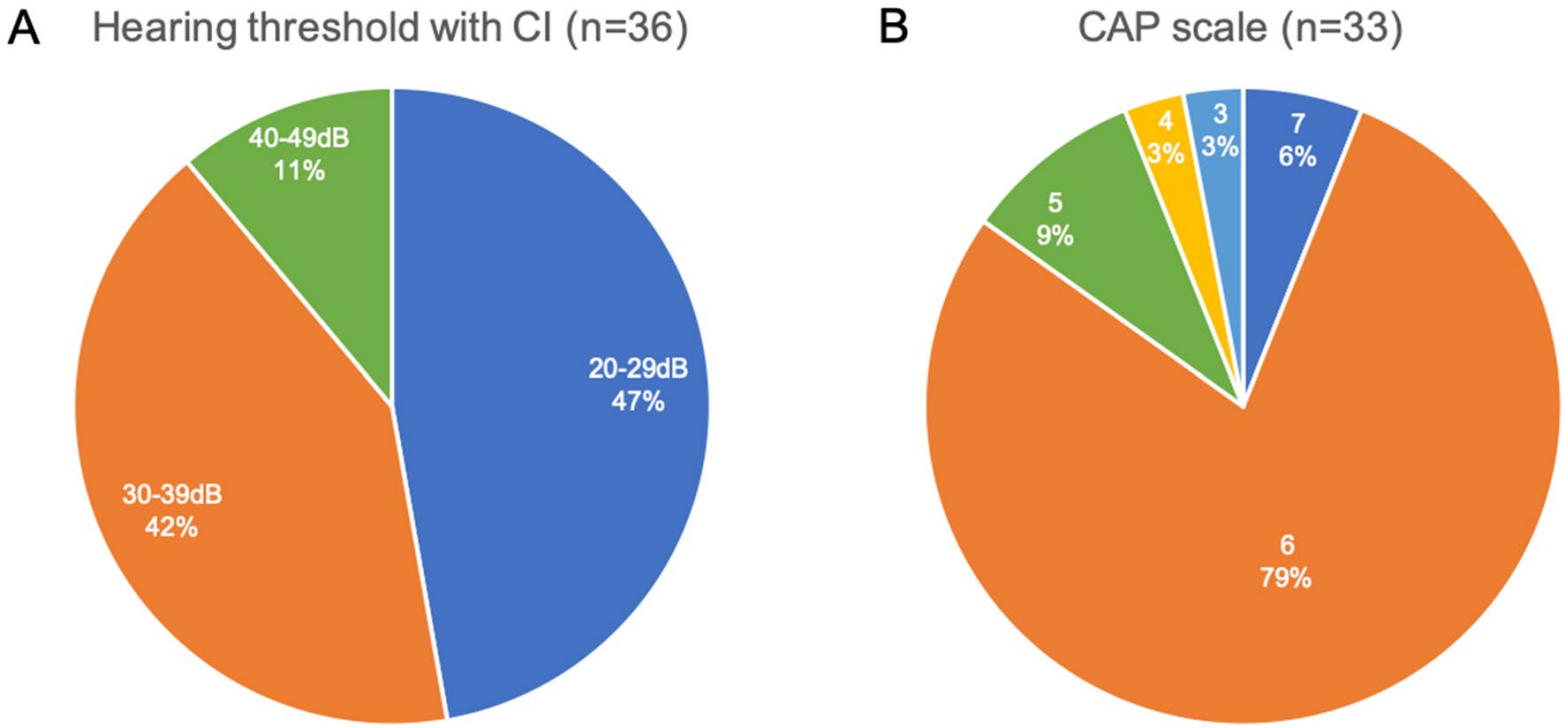
Performance of cochlear implantation for *OTOF*-related hearing loss. **a** Hearing threshold with cochlear implants was available for 36 patients who had used CIs for more than 2 years. **b** The CAP scale was available for 33 patients

In this study, the performance of cochlear implantation was evaluated using PTA and the Categories of Auditory Performance (CAP) scale for 36 patients who had used CI for over 2 years after the initial implantation. Cochlear implantations were performed unilaterally or bilaterally depending on the decisions made at each institution (Table 1). Hearing threshold data with CI in the better hearing ear were available for all of 36 patients and are shown in Fig. 3a: 20–29 dB in 17 patients (47.2%), 30–39 dB in 15 patients (41.6%) and 40–49 dB in 4 patients (11.1%). The CAP scale data were available for 33 patients and are shown in Fig. 3b: CAP scale level 7 in 2 patients (6.1%), level 6 in 26 patients (87.8%), level 5 in 3 patients (9.1%), level 4 in 1 patient (3%) and level 3 in 1 patient (3%).

### Disappearance of OAE

**Fig. 4:**
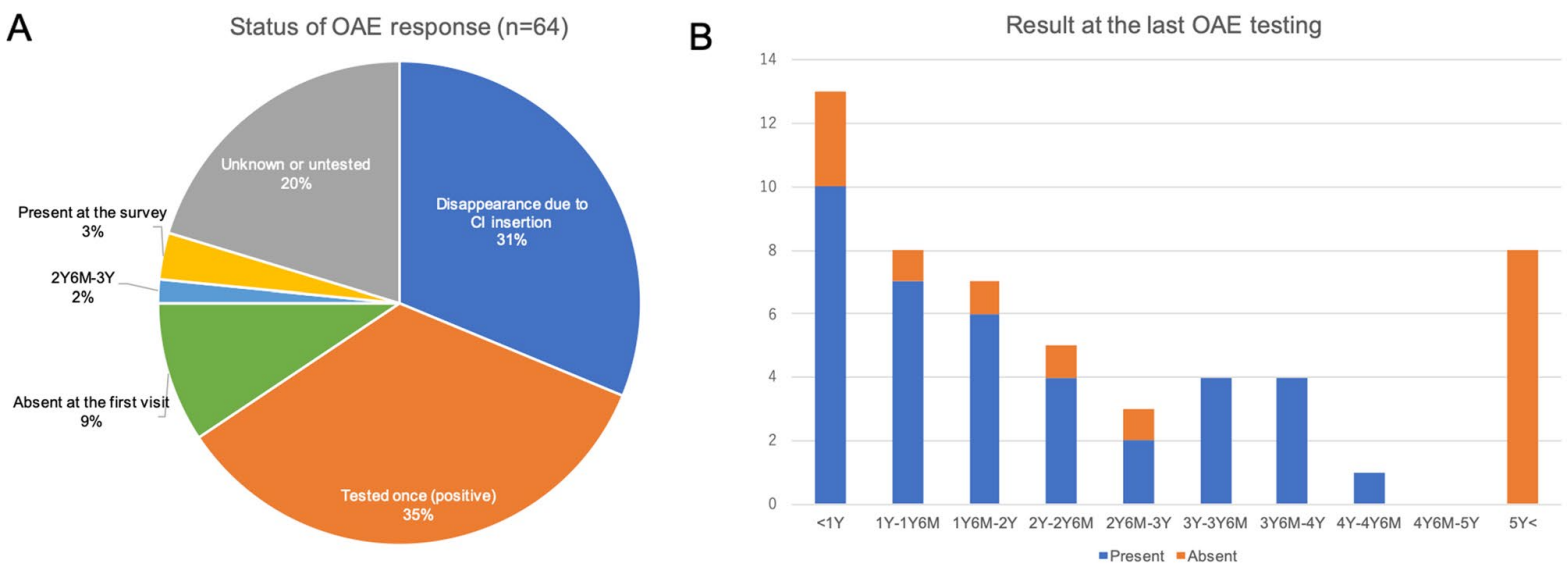
Detailed information on the disappearance of OAE response. **a** Timing of the disappearance of OAE response in the ear in which OAE response remained longer or the current status of OAE response. **b** Results at the last OAE testing

Figure 4A shows the timing of the disappearance of OAE response in the ear in which the OAE response remained longer; 20 patients (30.8%) lost OAE response at CI implantation, 22 patients (33.8%) were tested only once and showed OAE, 6 patients (9.2%) showed no response at the first visit and 2 patients (3.1%) still had a positive OAE at the time of this survey. Accurate timing of OAE disappearance was available only in one patient (between two and half years to three years old). Figure 4b shows the OAE results for the ears in which OAE response remained longer at the last OAE testing. No patients showed OAE passed 5 years of age.

## Discussion

**Fig. 5:**
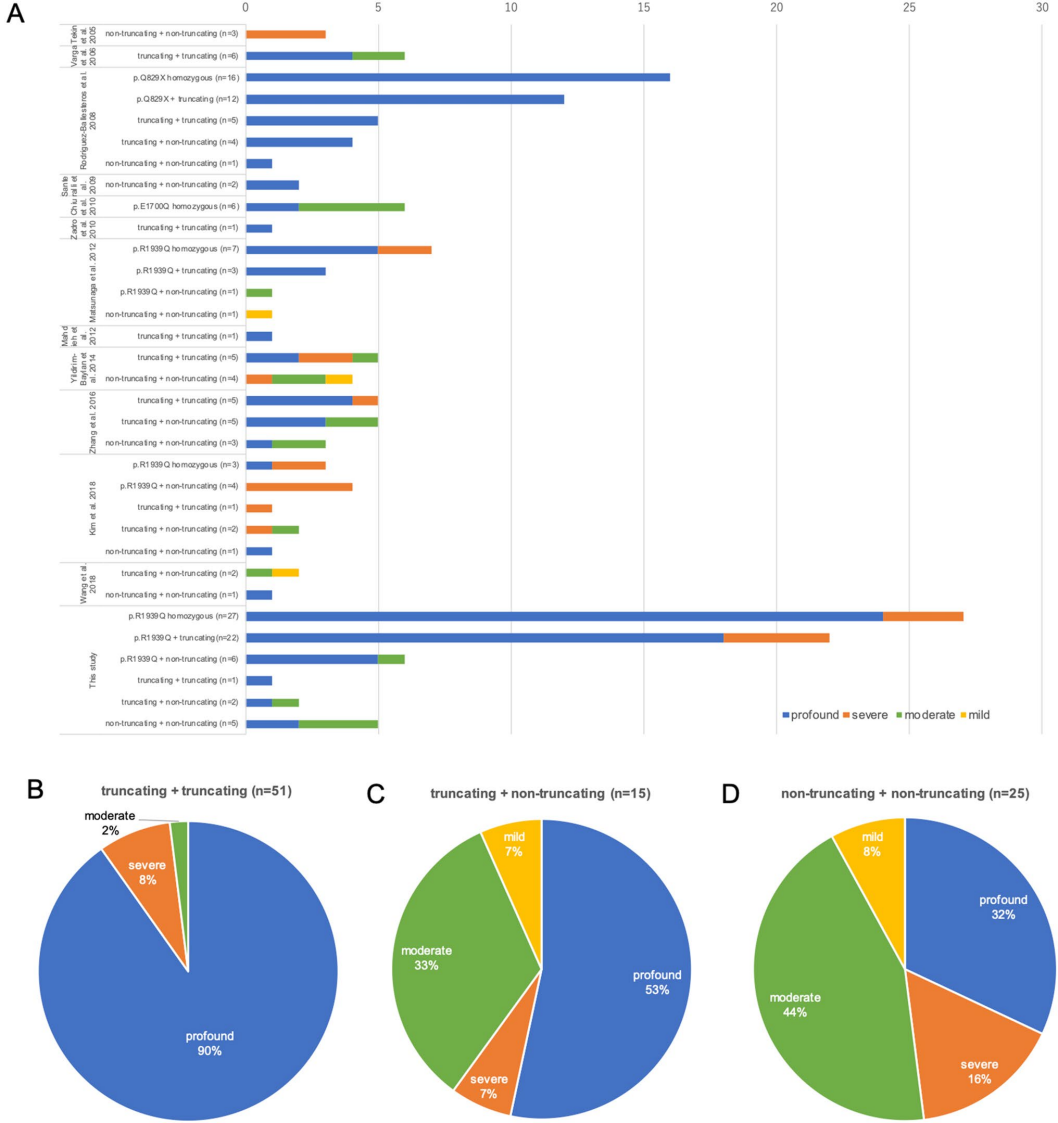
Summary of genotype–phenotype correlations in this and previous studies. **a** Mutation type and hearing severity in this and previous studies. **b** Hearing severity of patients with two truncating mutations. **c** Hearing severity of patients with truncating and non-truncating mutations (excluding p.Arg1939Gln). **d** Hearing severity of patients with two non-truncating mutations (excluding p.Arg1939Gln)

In this study, we investigated the clinical characteristics of 64 patients who were diagnosed with *OTOF*-related hearing loss. One of the main aims was to identify genotype–phenotype correlations. As reported previously, most of the patients (90.6%) participating in this study showed a “typical” phenotype; congenital or prelingual onset and severe-to-profound hearing loss (Fig. 1). All of the patients who revealed a homozygous c.5816G > A (p.Arg1939Gln) and compound heterozygote with p.Arg1939Gln and a truncating mutation, and 1 patient with two truncating mutations (c.1422 T > A (p.Tyr474Ter) and c3126 + 5G > A) showed profound hearing loss. Therefore, it is plausible that p.Arg1939Gln and a truncating mutation are related to severe-to-profound hearing loss based on these results. On the other hand, the phenotype of patients with non-truncating mutations is more complicated. Figure 5a shows a summary of genotype–phenotype correlations in this and previous studies, in which the patients’ hearing level and mutation information were available (Tekin et al. 2005; Varga et al. 2006; Rodríguez-Ballesteros et al. 2008; Santarelli et al. 2009; Chiu et al. 2010; Zadro et al. 2010; Mahdieh et al. 2012; Matsunaga et al. 2012; Yildirim-Baylan et al. 2014; Zhang et al. 2016; Kim et al. 2018; Wang et al. 2018). There is a clear relationship between either a homozygous or compound heterozygous state with p.Arg1939Gln and a severe phenotype. Truncating mutations including c.2485C > T (p.Gln829Ter) are also clearly related to a severe phenotype. Among patients having one or more non-truncating mutation, almost half showed mild-to-moderate hearing loss (Fig. 5c, d), even when possessing p.Arg1939Gln or a truncating mutation on the second allele. In cases with non-truncating mutations, the severity of clinical phenotype appears to vary depending on the functional alterations caused by each mutation. For instance, p.Arg1939Gln is clearly related to a severe phenotype according to clinical evidence to date, suggesting that the impact caused by the mutation strongly affects the function of otoferlin. However, no functional analysis has been reported, as with the most of other mutations. Genotype–phenotype correlation in non-truncating mutations remains partially unclear: the same mutation can cause different phenotypes, suggesting that other factors may affect the phenotype, such as mutations in other genes or epigenetic changes. Further research is needed on the issue.

Among the moderate cases in our cohort, there were three representative cases who showed “true” auditory neuropathy-like clinical characteristics (AK6640, AL8610 and AH0083 were compound heterozygote with c.[5816G > A];[1538A > G] (p.[Arg1939Gln];[His513Arg], c.[5815C > T];[5728G > A](p.[Arg1939Trp];[Glu1910Lys]) and c.[4718 T > C];[4129_4138del](p.[Ile1573Thr];[Ala1377Argfs*142], respectively)); the PTA of all patients showed moderate hearing loss while their ABR showed no response. p.His513Arg is located near the p.Ile515Thr and p.Gly541Ser mutations in the C2C domain, which are reported as mutations related to temperature-sensitive auditory neuropathy (TS-AN) and milder hearing loss in a non-febrile state (Varga et al. 2006; Matsunaga et al. 2012). *Otof*^Ile515Thr/Ile515Thr^ mice exhibited moderate hearing impairment with lower otoferlin levels and enlarged synaptic vesicles in the IHCs (Strenzke et al. 2016), suggesting that mutations around the area in the C2C domain may cause a milder phenotype. p.Ile1573Thr was reported as the cause of ANSD with mild-to-moderate hearing loss in a previous report (Yildirim-Baylan et al. 2014), which is compatible with the present case. We consider that p.His513Arg and p.Ile1573Thr are related to a milder phenotype. p.Arg1939Trp was reported previously, and the patient with homozygous p.Arg1939Trp showed severe-to-profound hearing loss (Choi et al. 2009). p.Glu1910Lys is novel mutation. As p.Arg1939Trp causes the same amino acid change as p.Arg1939Gln, p.Glu1910Lys might cause moderate hearing loss. Other than these mutations, it has been reported that p.Glu1700Gln was related to a progressive and milder phenotype (Chiu et al. 2010), and p.Arg1607Trp and p.Gly1804del were related to TS-AN (Marlin et al. 2010; Wang et al. 2010a). Interestingly, all of the patients with the aforementioned mutations related to a milder and non-typical phenotype showed no ABR response, just like “true” auditory neuropathy. Electrophysiological testing, such as ABR, is important, especially when estimating the hearing threshold of very young children. However, when determining the hearing level for *OTOF*-related hearing loss, the results of audiological testing should be carefully interpreted, considering that there is a discrepancy between PTA threshold and ABR response, especially when the PTA thresholds are mild-to-moderate.

Different mutation spectra and recurrent mutations have been reported in each population. p.Gln829Ter is quite frequently found in the Spanish population, being present in about 3% of all cases of recessive prelingual deafness (Migliosi et al. 2002). c.2905_2923delinsCTCCGAGCGGCA in Argentinean (Rodríguez-Ballesteros et al. 2008), p.Val1778Phe in Ashkenazi Jewish (Fedick et al. 2016), p.Glu57Ter and p.Arg1792His in Saudi Arabian (Almontashiri et al. 2018) and p. Glu1700Gln in Taiwanese populations (Chiu et al. 2010) are also common mutations in the respective populations as well. In this study, 59 of 65 patients (90.8%) had at least one p.Arg1939Gln mutation, which was reported to be a founder mutation (Matsunaga et al. 2012), suggesting that it is indeed a recurrent mutation in Japanese.

This study revealed that the detection rate of *OTOF*-related hearing loss in NBHS was 65.9% among the 44 patients for whom NBHS information was available in this study, which means one-third of *OTOF*-related hearing loss patients improperly pass screening in Japan. The presence of OAE is the main reason for missing *OTOF*-related ANSD at NBHS. Similar to our result, it was reported that 75% of *OTOF*-related patients improperly passed NBHS in a previous study (Wu et al. 2019). The risk of using OAE for NBHS has been discussed from the point of view of its inability to detect ANSD. Nevertheless, OAE is still used for NBHS in most countries and there are only a few countries where both OAE and AABR are used for NBHS (Wroblewska-Seniuk et al. 2017). In Japan, as is in some other countries, the screening method differs depending on the clinic and prefecture. All the patients in this study screened by OAE passed NBHS improperly, and their hearing loss was detected by their unawareness to sound or delayed language development. For better language development, early detection of HL is important. We strongly believe that NBHS should be performed by AABR or combination of OAE and AABR for reliable detection of ANSD.

The efficacy of cochlear implantation for ANSD has been controversial due to the heterogenicity of ANSD in terms of its etiology: ANSD is a disorder which includes auditory synaptopathy and neuropathy. Generally, prior to cochlear implantation, genetic testing should be considered to clarify which part of the auditory pathway is impaired and in order to be able to predict the outcomes of CI (Miyagawa et al. 2016). Once a patient is diagnosed with *OTOF*-related ANSD, a good CI outcome is expected because the auditory nerve remains intact. Indeed, past reports suggested that patients with *OTOF*-related ANSD are good candidates for CI (Zheng and Liu 2020). In this study, we investigated the efficacy of CI for *OTOF*-related hearing loss in the biggest cohort studied to date. Most of the patients who underwent cochlear implantation showed successful outcomes: approximately 85–90% of patients showed a hearing level of 20–39 dB with CI and a CAP scale level 6 or better, which means the *OTOF*-related hearing loss patients who underwent CI can understand conversation without lip reading. These data will support preoperative counseling for *OTOF*-related ANSD patients who are considered to be CI candidates.

In this study, we also investigated the timing of OAE disappearance. Although the exact timing of OAE disappearance was not available for most of the patients, some patients showed a positive OAE even at 3–4 years of age (Fig. 3b). Given no patients showed a positive OAE passed 5 years of age, we presume that the OAE response in *OTOF*-related ANSD disappears by 4–5 years of age at most, a little longer than previously thought. Related to the disappearance of OAE, some reports suggest that the disappearance of OAE could occur due to OHC damage originating from hearing aid use (Rouillon et al. 2006; Vona et al. 2020). On the other hand, it is also suggested that the use of hearing aids is not a definitive factor for deterioration of OAE (Kitao et al. 2019). In our study, some patients showed similar OAE responses even after wearing HAs until CI surgery, and some lost OAE response regardless of HA use; twenty patients lost OAE response due to CI, not due to wearing HAs (Fig. 4a), and three patients (AH9534, AG6481 and AK6678) lost OAE response under one year of age with or without a very short period wearing HAs (Fig. 4b). Therefore, it is unclear whether the disappearance of OAE is related to wearing HAs or is part of the natural course of *OTOF*-related hearing loss. As early intervention of HA for deaf patients is important for language development, there is no evidence for not recommending HAs for patients with *OTOF*-related ANSD with the aim of maintaining OAE response.

Although CI provide excellent hearing performance in cases of *OTOF*-related ANSD, it does not reach normal hearing level. Therefore, there has been a strong desire for a curative therapy, such as gene therapy. Recently, two reports have shown that cochlear gene therapy mediated by adeno-associated virus (AAV) successfully improved the prognosis of hearing impairment in *Otof*^*−/−*^ mice (Al‐Moyed et al. 2018; Akil et al. 2019). A new era of next-generation treatments for hereditary hearing loss, such as gene therapy, is certainly approaching; however, we should not forget that accurate treatment is based on accurate diagnosis and understanding of the clinical course. With the improvements in molecular biological diagnostic method, the etiologies of hereditary hearing loss have gradually become better understood. Although genotype–phenotype correlations have been obscure, the accumulation of patient data is gradually revealing the clinical characteristics of *OTOF*-related hearing loss. We believe that the clinical characteristics and genotype–phenotype correlation found in this study will support appropriate intervention and future treatment for *OTOF*-related hearing loss patients.

## Supplementary Information

Below is the link to the electronic supplementary material.

Supplementary file1 (XLSX 14 KB)
