# Correction to: Detailed clinical features and genotype–phenotype correlation in an *OTOF*-related hearing loss cohort in Japan

**DOI:** 10.1007/s00439-021-02392-y

**Published:** 2021-11-02

**Authors:** Yoh-ichiro Iwasa, Shin-ya Nishio, Hidekane Yoshimura, Akiko Sugaya, Yuko Kataoka, Yukihide Maeda, Yukihiko Kanda, Kyoko Nagai, Yasushi Naito, Hiroshi Yamazaki, Tetsuo Ikezono, Han Matsuda, Masako Nakai, Risa Tona, Yuika Sakurai, Remi Motegi, Hidehiko Takeda, Marina Kobayashi, Chiharu Kihara, Takashi Ishino, Shin-ya Morita, Satoshi Iwasaki, Masahiro Takahashi, Sakiko Furutate, Shin-ichiro Oka, Toshinori Kubota, Yasuhiro Arai, Yumiko Kobayashi, Daisuke Kikuchi, Tomoko Shintani, Noriko Ogasawara, Yohei Honkura, Shuji Izumi, Misako Hyogo, Yuzuru Ninoyu, Mayumi Suematsu, Jun Nakayama, Nana Tsuchihashi, Mayuri Okami, Hideaki Sakata, Hiroshi Yoshihashi, Taisuke Kobayashi, Kozo Kumakawa, Tadao Yoshida, Tomoko Esaki, Shin-ichi Usami

**Affiliations:** 1 Department of Otorhinolaryngology, Shinshu University School of Medicine, Matsumoto, Japan; 2 Department of Hearing Implant Sciences, Shinshu University School of Medicine, 3-1-1, Asahi, Matsumoto City, 390-8621 Japan; 3 Department of Otolaryngology-Head and Neck Surgery, Okayama University Graduate School of Medicine, Dentistry and Pharmaceutical Sciences, Okayama, Japan; 4 Kanda ENT Clinic, Nagasaki Bell Hearing Center, Nagasaki, Japan; 5 TAKASAKI Ear Nose and Throat Clinic, Takasaki, Japan; 6 Department of Otolaryngology, Kobe City Medical Center General Hospital, Kobe, Japan; 7 Department of Otorhinolaryngology, Saitama School of Medicine, Moroyama, Japan; 8 Shiga Medical Center for Children, Shiga, Japan; 9 Department of Otorhinolaryngology, Jikei University School of Medicine, Tokyo, Japan; 10 Department of Otorhinolaryngology, Juntendo University Faculty of Medicine, Tokyo, Japan; 11 Department of Otorhinolaryngology, Toranomon Hospital, Tokyo, Japan; 12 Department of Otolaryngology-Head and Neck Surgery, Nagasaki University Graduate School of Biomedical Sciences, Nagasaki, Japan; 13 Department of Otorhinolaryngology, Head and Neck Surgery, Hiroshima University Hospital, Hiroshima, Japan; 14 Department of Otolaryngology-Head and Neck Surgery, Faculty of Medicine and Graduate School of Medicine, Hokkaido University, Sapporo, Japan; 15 Department of Otorhinolaryngology, International University of Health and Welfare, Mita Hospital, Tokyo, Japan; 16 Department of Otolaryngology, Head and Neck Surgery, Yamagata University Faculty of Medicine, Yamagata, Japan; 17 Department of Otorhinolaryngology-Head and Neck Surgery, Yokohama City University School of Medicine, Yokohama, Japan; 18 Department of Otolaryngology-Head and Neck Surgery, Iwate Medical University, Morioka, Japan; 19 Department of Otolaryngology, Fukushima Medical University, Fukushima, Japan; 20 Department of Microbiology, Sapporo Medical University School of Medicine, Sapporo, Japan; 21 Department of Otolaryngology-Head and Neck Surgery, Tohoku University School of Medicine, Sendai, Japan; 22 Department of Otolaryngology Head and Neck Surgery, Niigata University Graduate School of Medical and Dental Sciences, Niigata, Japan; 23 Department of Otolaryngology-Head and Neck Surgery, Kyoto Prefectural University of Medicine, Kyoto, Japan; 24 Department of Otorhinolaryngology, Shiga University School of Medical Science, Otsu, Japan; 25 Department of Otorhinolaryngology, Graduate School of Medical Sciences, Kyushu University, Fukuoka, Japan; 26 Department of Otorhinolaryngology, Tokai University School of Medicine, Isehara, Japan; 27 Kawagoe Otology Institute, Kawagoe, Japan; 28 Department of Medical Genetics, Tokyo Metropolitan Children’s Medical Center, Tokyo, Japan; 29 Department of Otolaryngology, Kochi University Medical School, Kochi, Japan; 30 Department of Otolaryngology, Kamio Memorial Hospital, Tokyo, Japan; 31 Department of Otorhinolaryngology, Nagoya University Graduate School of Medicine, Nagoya, Japan; 32 Department of Otolaryngology, Aichi Children’s Health and Medical Center, Obu, Japan

## Correction to: Human Genetics 10.1007/s00439-021-02351-7

In the original article published, there is a correction in Table 1. We would like to remove the variant (c.4023 + 1G > A) from the Table. The correct Table 1 is attached below.

**Table1:** Detailed mutational and clinical information for the cases with biallelic OTOF mutations in this study

Patient ID	Age	Mutation 1	Mutation 2	OAE*	ABR	Severity^†^	Intervention	CAP	Age at first CI
SNS2255	34y	p.Arg1939Gln	p.Arg1939Gln	Absent	Untested	Profound	Hearing aid	6	–
YMG2003	23y	p.Arg1939Gln	p.Arg1939Gln	NA	No response	Profound	Unilateral CI	6	5y–5y6m
2703	22y	p.Arg1939Gln	p.Arg1939Gln	NA	No response	Severe	Hearing aid	NA	–
AH6163	16y	p.Arg1939Gln	p.Arg1939Gln	NA	100–105 dB	Profound	Unilateral CI	6	6y <
SNS1171	13y	p.Arg1939Gln	p.Arg1939Gln	Present	NA	Profound	Bilateral CI (sequential)	6	1y6m–2y
AG7860	12y	p.Arg1939Gln	p.Arg1939Gln	NA	No response	Profound	Unilateral CI	6	NA
KBS5019	11y	p.Arg1939Gln	p.Arg1939Gln	Present	No response	Profound	Bilateral CI (sequential)	6	3y–3y6m
AG4113	9y	p.Arg1939Gln	p.Arg1939Gln	Present	No response	Profound	Hearing aid	3	–
OKY3003	9y	p.Arg1939Gln	p.Arg1939Gln	Present	100–105 dB	Profound	Bilateral CI (sequential)	6	1y–1y6m
SNS2292	9y	p.Arg1939Gln	p.Arg1939Gln	Present	No response	Profound	Bilateral CI (sequential)	6	1y–1y6m
AL8879	7y	p.Arg1939Gln	p.Arg1939Gln	Present	90–99 dB	Profound	Unilateral CI	5	3y6m–4y
AH1024	7y	p.Arg1939Gln	p.Arg1939Gln	NA	Untested	Profound	Bilateral CI (sequential)	6	1y6m–2y
AL8222	6y	p.Arg1939Gln	p.Arg1939Gln	Present	100–105 dB	Profound	Bilateral CI (sequential)	6	2y–2y6m
AK2367	5y	p.Arg1939Gln	p.Arg1939Gln	Present	90–99 dB	Profound	Bilateral CI (sequential)	6	1y–1y6m
AK2846	5y	p.Arg1939Gln	p.Arg1939Gln	Present	Untested	Profound	Bilateral CI (simultaneous)	7	1y–1y6m
AL8158	4y	p.Arg1939Gln	p.Arg1939Gln	Present	90–99 dB	Profound	Bilateral CI (sequential)	6	1y–1y6m
AK6678	4y	p.Arg1939Gln	p.Arg1939Gln	Present	No response	Profound	Bilateral CI (sequential)	6	1y6m–2y
AL5519	4y	p.Arg1939Gln	p.Arg1939Gln	Absent	No response	Profound	Bilateral CI (sequential)	5	1y6m–2y
AL7410	3y	p.Arg1939Gln	p.Arg1939Gln	Present	40–49 dB	Profound	Bilateral CI (simultaneous)	6	1y–1y6m
AM9889	3y	p.Arg1939Gln	p.Arg1939Gln	Present	100–105 dB	Profound	Bilateral CI (simultaneous)	6	1y6m–2y
AH0075	3y	p.Arg1939Gln	p.Arg1939Gln	Present	100–105 dB	Severe	Bilateral CI (simultaneous)	3	1y–1y6m
AM7901	2y	p.Arg1939Gln	p.Arg1939Gln	Present	No response	Profound	Unilateral CI	5	2y6m–3y
AM8170	2y	p.Arg1939Gln	p.Arg1939Gln	Present	No response	Profound	Unilateral CI	1	2y–2y6m
AK2888	1y	p.Arg1939Gln	p.Arg1939Gln	NA	No response	Profound^‡^	Bilateral CI (simultaneous)	NA	NA
HL9490	1y	p.Arg1939Gln	p.Arg1939Gln	Present	No response	Profound^‡^	Hearing aid	3	–
AK6261	1y	p.Arg1939Gln	p.Arg1939Gln	NA	Untested	Profound^‡^	Hearing aid	3	–
AL7917	1y	p.Arg1939Gln	p.Arg1939Gln	Present	100–105 dB	Severe^‡^	Hearing aid	3	–
AH1616	70y	p.Arg1939Gln	p.Tyr474Ter	NA	Untested	Severe	NA	4	–
AK9968	41y	p.Arg1939Gln	c.5533 + 1G > A	Absent	NA	Profound	Hearing aid	4	–
AH2665	32y	p.Arg1939Gln	p.Tyr474Ter	NA	Untested	Profound	Hearing aid	4	–
2958	20y	p.Arg1939Gln	p.Trp717Ter	Present	NA	Severe	Unilateral CI	6	3y6m–4y
AH6310	11y	p.Arg1939Gln	p.Gln1072Ter	NA	Untested	Profound	Bilateral CI (sequential)	7	1y6m–2y
AH1786	10y	p.Arg1939Gln	p.Tyr474Ter	Present	Untested	Profound	Bilateral CI (sequential)	6	1y6m–2y
AG6001	9y	p.Arg1939Gln	p.Tyr1064Ter	Present	No response	Profound	Bilateral CI (sequential)	6	1y–1y6m
AH8883	8y	p.Arg1939Gln	p.Pro489Ser	Present	No response	Profound	Bilateral CI (sequential)	6	2y6m–3y
AG9191	8y	p.Arg1939Gln	p.Arg1792Cys	Present	No response	NA	NA	NA	NA
AH9534	7y	p.Arg1939Gln	c.4960 + 2T > C	Absent	No response	Profound	Bilateral CI (sequential)	6	1y6m–2y
AH0904	7y	p.Arg1939Gln	p.Ile1449fs	Present	No response	Severe	Unilateral CI	6	1y–1y6m
AH6306	6y	p.Arg1939Gln	p.Gln1072Ter	Present	No response	Profound	Bilateral CI (sequential)	6	1y6m–2y
D72	6y	p.Arg1939Gln	p.Tyr1064Ter	Present	No response	Profound	Bilateral CI (sequential)	6	< 1y
AG6481	6y	p.Arg1939Gln	p.Arg1856Gln	Absent	No response	Profound	Bilateral CI (sequential)	4	1y–1y6m
AL8880	6y	p.Arg1939Gln	p.Arg1939Trp	Present	No response	Profound	Unilateral CI	6	2y–2y6m
AH0951	5y	p.Arg1939Gln	p.Tyr474Ter	Present	No response	Profound	Unilateral CI	3	1y–1y6m
AK6640	5y	p.Arg1939Gln	p.His513Arg	Untested	No response	Moderate	not-aided	6	–
AG6165	5y	p.Arg1939Gln	p.Leu1003fs	Present	NA	Profound	Bilateral CI (simultaneous)	6	1y–1y6m
AL7878	5y	p.Arg1939Gln	p.Arg897fs	Present	No response	Profound	Hearing aid	NA	–
AK2019	5y	p.Arg1939Gln	p.Arg1856Trp	Present	No response	Profound	Unilateral CI	6	NA
AL7890	4y	p.Arg1939Gln	p.Arg1939Trp	Present	90–99 dB	Profound	Bilateral CI (sequential)	6	2y6m–3y
AP8312	4y	p.Arg1939Gln	p.Glu757fs	Present	No response	Profound	Bilateral CI (sequential)	5	2y–2y6m
AM8924	3y	p.Arg1939Gln	p.Tyr474Ter	Present	No response	Profound	Bilateral CI (simultaneous)	6	1y–1y6m
AL7003	3y	p.Arg1939Gln	c.897 + 5G > A	Absent	No response	Profound	Bilateral CI (sequential)	4	1y–1y6m
AL6924	2y	p.Arg1939Gln	p.Tyr474Ter	Present	NA	Profound	Bilateral CI (simultaneous)	NA	NA
AM9635	2y	p.Arg1939Gln	p.Gln1072Ter	Present	No response	Profound	Bilateral CI (simultaneous)	0	2y–2y6m
AL7907	2y	p.Arg1939Gln	p.Trp1946Ter	Present	No response	Profound	Bilateral CI (sequential)	5	1y–1y6m
AL6094	2y	p.Arg1939Gln	p.Asp1834fs	Present	100–105 dB	Profound	Bilateral CI (sequential)	4	2y–2y6m
AL9858	1y	p.Arg1939Gln	c.3570 + 5G > A	Present	Untested	Severe‡	Hearing aid	NA	–
AL5758	18y	p.Arg1727Gln	p.1123_1129del	Absent	60–69 dB	Moderate	not-aided	NA	–
KND0018	17y	p.Arg1856Gln	p.Tyr474Ter	Present	No response	Profound	Unilateral CI	6	NA
AK3328	16y	p.Tyr474Ter	c.3126 + 5G > A	Absent	Untested	Profound	Hearing aid	3	–
AR7215	12y	p.Arg1939Trp	p.Glu1910Lys	Present	No response	Profound	Unilateral CI	NA	6y <
AK2839	10y	p.Ser247Asn	p.Glu594Lys	Untested	No response	Profound	Bilateral CI (sequential)	NA	2y6m–3y
KBS5114	9y	p.Phe1069Val	p.Ala1802Val	Untested	Untested	Moderate	Hearing aid	6	–
AL8610	9y	p.Arg1939Trp	p.Glu1910Lys	Present	No response	Moderate	Hearing aid	NA	–
AH0083	7y	p.Ile1573Thr	p.Ala1377fs	Present	No response	Moderate	Unilateral CI	6	6y <

*NA* not available, *m* month(s), *y* year(s)

*OAE responses are based on the results at NBHS or first testing at each institution

^†^Theresholds were determined as the average at 500, 1000, 2000 and 4000 Hz in the better hearing ear tested by PTA or COR

^‡^Hearing level tested by COR

The original article has been corrected.

